# One health approach to toxocariasis in quilombola communities of southern Brazil

**DOI:** 10.1186/s13071-023-06010-w

**Published:** 2023-10-23

**Authors:** Vamilton Alvares Santarém, Giovanni Kalempa Panazzolo, Louise Bach Kmetiuk, Orlei José Domingues, Isabella Braghin Ferreira, Roberto Teixeira de Souza Filho, João Henrique Farinhas, Fernando Rodrigo Doline, Susana Angélica Zevallos Lescano, Leandro Meneguelli Biondo, Rogério Giuffrida, Alexander Welker Biondo, Giovani Marino Fávero

**Affiliations:** 1 Graduate College of Animal Science, University of Western São Paulo, Presidente Prudente, São Paulo Brazil; 2 Graduate College of Pharmaceutical Sciences, State University of Ponta Grossa, Ponta Grossa, Paraná Brazil; 3 Graduate College of Cellular and Molecular Biology, Federal University of Paraná, Curitiba, Paraná Brazil; 4 Institute of Tropical Medicine of São Paulo, University of São Paulo, São Paulo, Brazil; 5 Brazilian Ministry of Science, Technology, and Innovation, National Institute of the Atlantic Forest (INMA), Santa Teresa, Espírito Santo Brazil; 6 Interdisciplinary Graduate Studies, University of British Columbia, Kelowna, BC Canada

**Keywords:** Epidemiology, Poverty, Quilombo, Seroprevalence, *Toxocara* spp., Zoonosis

## Abstract

**Background:**

Toxocariasis has been listed among the most neglected parasitic diseases worldwide, with approximately one fifth of the global population exposed, particularly those living under poverty. In Brazil, communities of descendants of enslaved blacks (quilombola) have historically had some of the highest rates of vulnerability and poverty, characterized by lack of health assistance, poor quality of life, and nutritional insecurity.

**Methods:**

A cross-sectional sampling of quilombola individuals living in four communities of southern Brazil, as well as their dogs and the soil, was carried out from December 2021 to March 2022. Sociodemographic and other information such as water source, alimentary habits, and dog and cat ownership were gathered using a semi-structured questionnaire for assessing toxocariasis risk factors. Human serum samples were tested by ELISA for anti-*Toxocara* spp. IgG antibody detection was carried out on dog feces and hair, and soil samples were surveyed for presence of *Toxocara* spp. eggs.

**Results:**

Overall, 172/208 individuals (82.7%, 95% CI = 77.0–87.2) were seropositive, the highest seroprevalence rate to date in Brazil. Male gender *(P* = 0.029), educational level *(P* = 0.026), and drinking water source *(P* = 0.043) were associated with seropositivity by univariate analysis. Final logistic regression revealed increased odds (*P* = 0.017, OR = 7.6, 95% CI = 1.5–42.7) to have seropositivity in individuals > 50 years old (< 10 years old). As expected, individuals with soil contact were more likely seropositive (*P* = 0.038, OR = 4.4, 95% CI = 1.1–18.8). Although retrieved in only 5/96 (5.2%) dog feces, *Toxocara* spp. eggs were found in 18/60 (30.0%) soil samples.

**Conclusions:**

The high vulnerability and seroprevalence observed in quilombola communities clearly demand a One Health approach for detection, monitoring, and prevention of infection by *Toxocara* spp. in both human and dog populations.

**Graphical Abstract:**

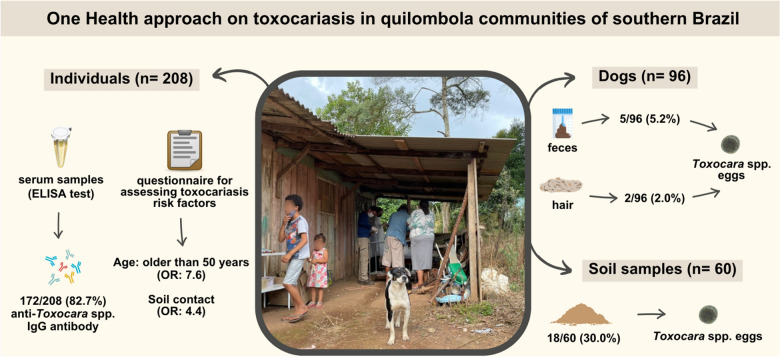

**Supplementary Information:**

The online version contains supplementary material available at 10.1186/s13071-023-06010-w.

## Background

Toxocariasis has been a prevalent zoonosis with a significant socioeconomic impact, particularly on impoverished communities worldwide [1], and has been included among the six most important neglected parasitic infections in the USA along with Chagas disease, cyclosporiasis, cysticercosis, toxoplasmosis, and trichomoniasis [2]. Characterized by significant seroprevalence and chronic disease and associated with poverty [3], this disease has also been considered the fourth and, perhaps equally important, a soil-transmitted helminth infection along with ascariasis, trichuriasis, and hookworm [4].

Accidental ingestion of soil containing *Toxocara canis* or *Toxocara cati* infective eggs has been considered the primary transmission route of human toxocariasis [5]. As natural definitive hosts, dogs and cats may shed roundworm eggs in feces, become infective following an embryonation period of 2 to 5 weeks, and persist in the environment for months [6]. Although some studies in Brazil have observed a higher recovery of eggs from soil in public parks in winter and summer [7], and throughout the year [8], to our knowledge no study assessing the persistence of *Toxocara* spp. eggs in tropical environments over time has been conducted to date.

Migration or larva-induced immune response may lead to several clinical manifestations in humans, with visceral toxocariasis mostly resulting in hepatic and pulmonary disorders [9]. The ocular form has reportedly caused visual impairment and blindness, while neurological presentation (neurotoxocariasis) may cause central nervous system impairment [10].

Approximately one fifth of the global human population has been exposed to toxocariasis agents according to a meta-analysis study, presenting the highest seroprevalence in Africa and Latin America [11]. Associated risk factors to *Toxocara* spp. seropositivity revealed higher risk of young people, males, those living in rural areas, those in close contact with dogs, cats, or soil, those consuming raw meat, and those drinking untreated water [11]. In Brazil, the highest seroprevalence to date (247/344; 71.8%) was observed in adult inhabitants of rural southern Brazil [12]. As social vulnerability has also been associated with toxocariasis, different vulnerable Brazilian populations have demanded a One Health approach and required simultaneous human-animal-soil samplings to address zoonotic issues in animal keepers and the homeless, incarcerated, indigenous, and traditional island populations [13].

**Fig. 1:**
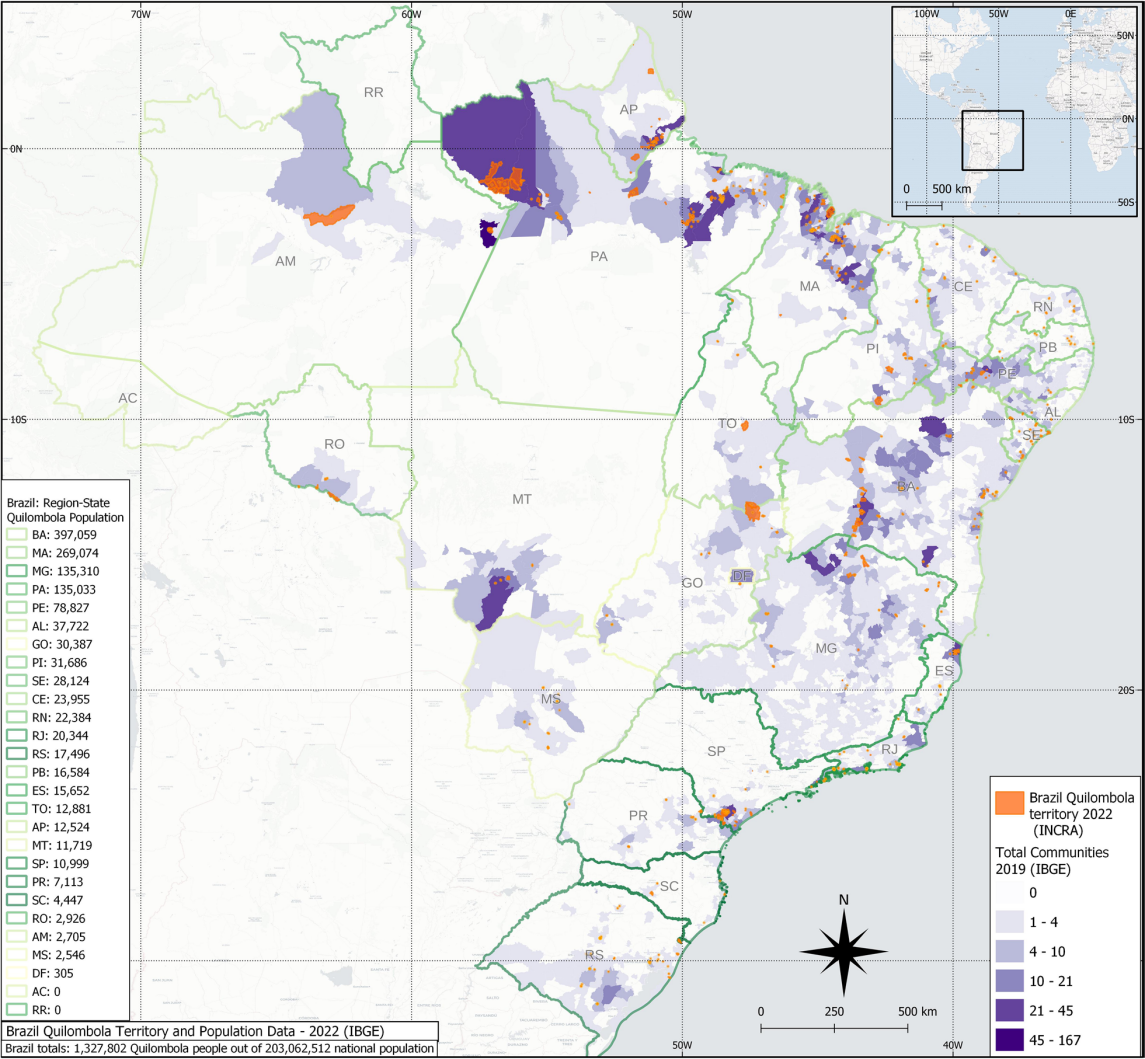
Quilombola communities, territories, and population in Brazil, according to the Brazilian Institute of Geography and Statistics (IBGE)

Another vulnerable and traditional Brazilian community (named quilombola) comprises former African slaves and their descendants, who have historically remained in rural and semi-isolated areas since the time of slavery [14], preserving their African culture and subsisting on agriculture and forest resources [15]. Despite historical government efforts, this population has lived under poverty, greater social and health needs, poor quality of life, and nutritional insecurity [16]. A total of 494 quilombola communities were registered in the recent 2022 census by the Brazilian Institute of Geography and Statistics (IBGE), with 167,202/1,327,802 (12.6%) individuals living within these territories out of the total of self-declared quilombola population in Brazil (Fig. 1) [17]. Quilombola communities have mostly dogs as companion animals, usually wandering but also including owned restricted and semi-restricted ones, which may carry zoonotic pathogens including *Ehrlichia* spp., *Anaplasma* spp., *Leishmania* spp., *Borrelia burgdorferi* s.l., and *Dirofilaria immitis* [18].

Besides social vulnerability, a favorable scenario for *Toxocara* spp. transmissionin quilombola communities may also include close and daily contact with agricultural labor and presence of untreated dogs, as dogs and cats in low-income and rural regions have played an important role in toxocariasis [5]. Nonetheless, to our knowledge no comprehensive study has been conducted in this susceptible population, particularly regarding a One Health approach. Accordingly, this study aimed to survey and analyze risk factors associated with human toxocariasis, including the presence of *Toxocara* spp. eggs in dog hair and feces and in soil samples in four rural quilombola communities of southern Brazil.

## Methods

### Study design

The present study was a cross-sectional survey conducted in four Brazilian quilombola (out of 34) communities located in Paraná State, southern Brazil, from December 2021 to March 2022 (Table 1). The study area herein included natural and degraded areas of two Brazilian biomes (Atlantic Forest and Cerrado), with humid temperate climate and averaging 17.5 °C in temperature and 1495 mm^3^ in annual precipitation. The land use and occupations in traditional quilombola communities have often been connected to a common use approach of territory. However, surrounding properties usually have not followed the same land use pattern, leading to conflicts and influencing the changes in perspective of what land means for members of traditional communities [19, 20]. Quilombola communities of Paraná State have lived mostly on subsistence agriculture and livestock, with continuous land struggle caused by surrounding commercial wood and cellulose production companies, mostly of *Eucalyptus* spp. trees.

**Table 1:** Characteristics of four quilombola communities located in rural areas of Paraná State, southern Brazil, for assessing risk factors of toxocariasis

Quilombola communities	Geographic coordinates	Estimated population*	*n* sampled (%)
Limitão	24°40′37.04′′S 49°35′53.05′′O	106	45 (42.5)
Mamans	24°55′11.73′′S 49°41′4.78′′O	48	42 (87.5)
Serra do Apon	24°51′45.40′′S 50°2′42.97′′O	145	70 (48.3)
Tronco	24°51′21.0′′S 50°01′49.0′′O	62	51 (82.3)

^*^Priori et al. [21]

The human sample size was calculated considering a 27.6% toxocariasis seropositivity in the general Brazilian population, based in a recent worldwide meta-analysis study [11], and an estimated population of 3800 quilombola individuals in the Paraná State [21]. An assumption of 6% precision was considered in an expected range prevalence of 10–90%, based on preliminary studies with 95% confidence level [22, 23], resulting in a minimum of 203 individuals.

A structured epidemiological questionnaire was applied, after the subjects provided an individual signed consent form, which included information on sociodemographic aspects such as age, gender, educational level, and potential associated risk factors such as the drinking water source or type, contact with soil, raw meat intake, ingestion of game meat, onychophagy, and owning dogs, cats, or both. Parents and other relatives provided written consent for subjects < 18 years old and illiterate individuals prior to blood collection and epidemiological questionnaire. In addition, dog owners were interviewed on the epidemiological questionnaires about themselves and their dogs and provided a signed consent form for blood samplings from them and their dogs.

Blood samples were conveniently collected from quilombola individuals by peripheral venipuncture, performed by a certified physician, nurse, or pharmacist and using commercial vacuum tubes, after providing a signed consent form. Samples were centrifuged at 1295 × *g* for 5 min and the obtained serum stored at −20 °C until processing.

### Detection of anti-*Toxocara* spp. IgG

#### Antigen preparation

In vitro production of *T. canis* excretory-secretory larval antigens (TES) was based on the method described by Savigny et al. [24], with some modifications [25].

#### Pre-adsorption of human serum

Serum samples were pre-adsorbed with *Ascaris suum* adult worm extract (AWE) following an established protocol [25] to remove antibodies elicited by exposure to *Ascaris* that could cross-react with *Toxocara* spp. antigens and, consequently, enhance the specificity of the ELISA test [26].

#### ELISA test

Serum samples were tested for IgG antibodies to TES by ELISA (enzyme-linked immunosorbent assay) at a dilution of 1:200 [25] at the Laboratory of Medical Investigation, Institute of Tropical Medicine of São Paulo, University of São Paulo, Brazil. The negative sera controls were stored in the serum bank and have been routinely used in ELISA for toxocariasis, with 78.3% sensitivity and 92.3% specificity, as previously reported in other studies [27, 28]. The sera were previously tested by an established protocol [25] and were negative for parasites in previous studies [29, 30].

Absorbance readings were made at 492 nm (Titertek Multiskan MCC/340, Lab-System, Finland), and a cut-off absorbance value was defined as the mean absorbance reading for 90 negative control sera plus three standard deviations. Standard positive and negative control serum and a threshold reactive serum were used in all tests. The antibody levels were expressed as reactivity indices (RIs) that were calculated as the ratio between the absorbance values of each test sample and the cutoff value, set at 0.350. A serum sample was considered positive when its RI was > 1.

#### Dog samples

Dog samples included feces and hair and were individually collected from 96 dogs living in the studied quilombola communities. Dogs were physically restrained; feces were collected from the rectum and transferred to 50-ml plastic tubes with 10% formalin solution to preserve parasite eggs [31]. Hair samples were collected at the dorsal and perineal areas from each dog using commercial blades and placed in 50-ml plastic tubes. Fecal and dog hair samples were kept under refrigeration at 4 °C until processing and analysis.

Dog feces samples were processed to assess the presence of *Toxocara* spp. eggs by using a flotation technique (hypersaturated sodium chloride solution d = 1.20 g/cm^3^). Results were considered positive or negative.

Hair samples were processed using a previous protocol described elsewhere [32], with some modification [33]. Briefly, hair samples were rinsed with anionic detergent (Tween 80^®^), sieved (300, 212 and 38 μm), and centrifugated (5 min, 1090 ×*g*). Then, pellets were analyzed under a microscope (magnifications: 10 × and 40×).

*Toxocara* spp. eggs recovered from dog hair were classified according to criteria, as previously established [32]: embryonated eggs (containing larva); embryonating eggs (cellular division); viable (intact eggs with content); non-viable eggs (wall disruption).

### Soil sampling and testing

Around 50 g of soil samples was randomly collected from the commonly used areas at the four quilombola communities, including community centers and trails. The sampling sites were selected by convenience, and free-roaming dogs were present there. Grass areas and presence of feces were considered exclusion criteria for local soil collection. Samples were collected at a depth of 5 to 10 cm, transferred to a plastic bag, and stored under refrigeration (4 ºC) until processing. A total of 60 soil samples were collected, ranging from 10 to 20 samples per community.

Soil analysis followed the protocol described elsewhere [34, 35] with some modifications [13]. Briefly, 20 g of soil was submitted to a protocol of rinsing with anionic detergent (100 ml 5% Tween 80^**®**^), sieving procedure (300 μm, 212 μm, 90 μm, and 38 μm), and centrifugation (800 × g for 5 min). Following this, the sediment was subjected to a centrifuge-flotation technique (zinc sulfate solution d = 1.35 g/cm^3^). After a 5-min flotation period, 5 ml of the floating content was transferred to two other graduated tubes (2.5 ml each). Then, 10 ml distilled water was added, and another centrifugation (800 ×*g* for 5 min) performed. The washing process was repeated three times to remove the sulfate solution. Then, the entire sediment was analyzed under a microscope (magnifications: 10 × and 40 ×), and *Toxocara* spp. eggs recovered from soil were classified as described [32].

### Statistical analysis

Categorical variables were subjected to univariate analysis to select possible factors associated with seropositivity for *Toxocara* spp. in quilombola populations, considering up to 15.0% missing data. The association was tested using the Chi-square test or Fisher test when expected values were < 5.0, with estimates of odds ratio and 95% confidence intervals. Variables with a significance level < 0.2 were used as potential predictors of seropositivity for a multivariate logistic model with variable selection by the backward stepwise algorithm. The model was evaluated for the presence of collinearity by estimating the variance inflation factor (VIF) and the accuracy in predicting seropositivity by ROC curve. The receiver-operating characteristic (ROC) is a graphical tool commonly used to assess the performance and accuracy of predictive models, particularly binary classification models like logistic regression for risk factors in epidemiological surveys [36]. The AUC was estimated by integral calculus of the ROC curve. The result provided a single value summarizing the model's discriminatory power, varying between 0.5 (indicating absence of discriminatory power between seropositive and seronegative cases) and 1.0 (representing perfect discrimination between seropositive and seronegative cases). All analyses were conducted using R software v. 4.2.2 and auxiliary packages, considering a significance level of 5% [37, 38].

## Results

The studied population was composed by adults (77.4%; 161/208) ranging from 1 to 99 years old (median: 37), mostly females (126/208; 60.6%). Most participants declared having elementary school level education (128/208; 61.5%) and contact with soil (188/208; 90.4%). All the adult participants mentioned subsistence agriculture activities (Additional file 2).

**Fig. 2:**
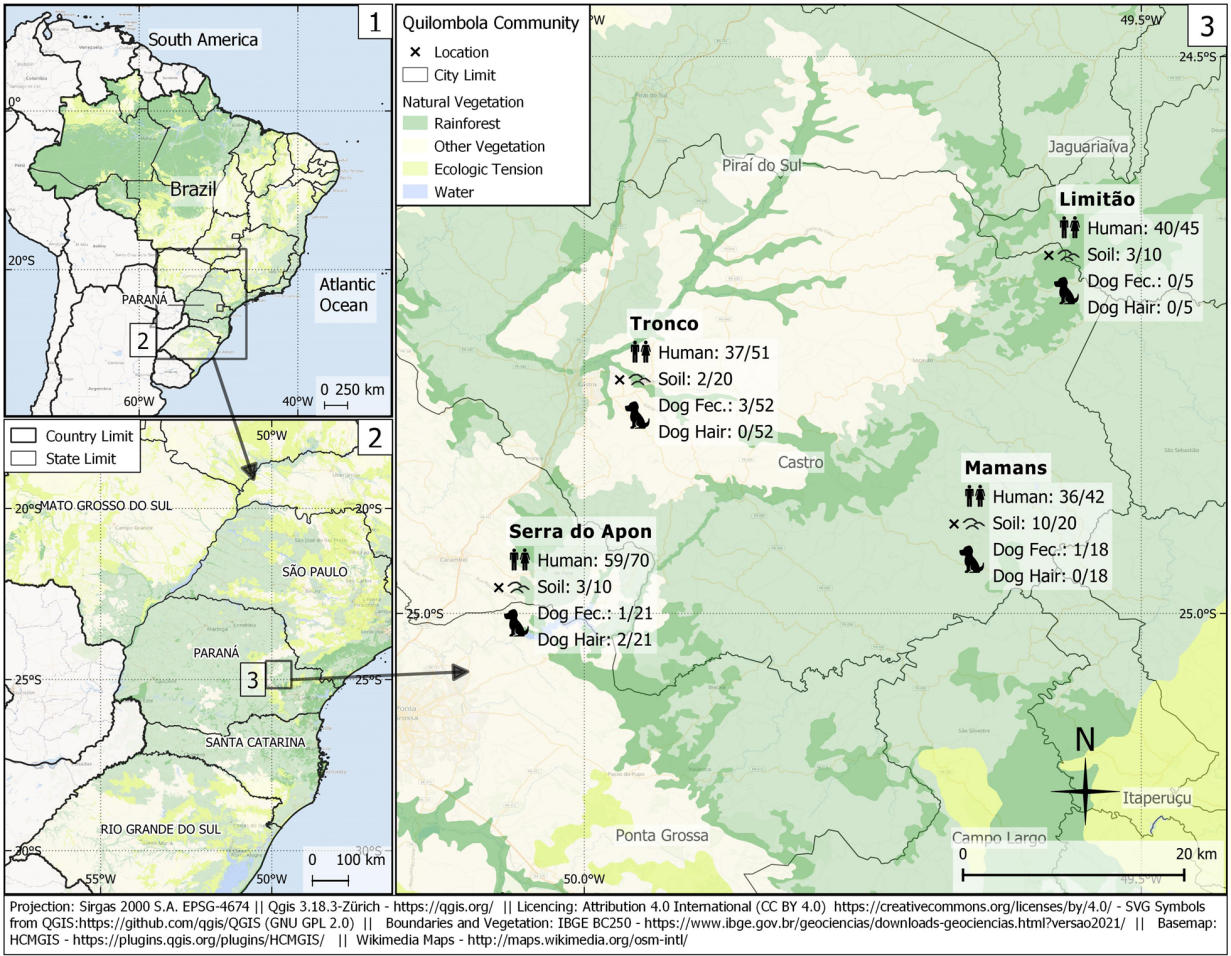
Sampling locations, frequency of occurrence of anti-*Toxocara* spp. antibodies in quilombola individuals, and the presence of feces, hair, and soil-positive samples in quilombola communities of southern Brazil

Overall, 172/208 (82.7%; 95% CI = 77.0–87.2) quilombola individuals were seropositive for circulating anti-*Toxocara* spp. antibodies (Fig. 2). Seroprevalence ranged from 72.6% to 88.9% in Tronco and Limitão communities, respectively (Table 2), with no statistical difference (*P* = 0.153) among frequencies. Associated Risk factors for the presence of anti-*Toxocara* spp. antibodies in participants by uni- and multivariate analysis were tested (Table 3), and five variables (gender, age, educational level, contact with soil, and drinking water source) were included in the multivariate logistic model. Logistic regression revealed only age and contact with soil as predictive variables for seropositivity. Increased odds *(P* = 0.017, OR = 7.6, 95% CI = 1.5–42.7) for seropositivity (Table 3) were observed in individuals aged > 50 years old compared to the reference group (< 10 years old) and in quilombola participants who had close contact with soil *(P* = 0.038, OR = 4.4, 95% CI = 1.1–18.8).

**Table 2:** Seroprevalence of anti-*Toxocara* spp. antibodies (IgG) detected by ELISA test in quilombola persons (*N *= 208) living in five communities of Paraná State, southern Brazil

Communities	Seropositivity/ subjects	Frequency (%)	95% CI
Limitão	40/45	88.9	76.5–95.2
Mamans	36/42	85.7	72.2–93.3
Serra do apon	59/70	84.3	74.0–91.0
Tronco	37/51	72.6	59.1–82.3
Total	172/208	82.7	77.0–87.2

**Table 3:** Univariate and logistic multivariate regression association between characteristics of quilombola communities (N = 208) and presence of anti-*Toxocara* spp. antibodies IgG, in communities of Paraná State, southern Brazil

Characteristics	Seropositive (%)	Seronegative (%)	Univariate analysis		Multivariate analysis	
	172 (82.7)	36 (17.3)	OR (95% CI)	*p*-value	OR (95% CI)	*p*-value
Gender				0.029*		
Female	98 (56.5)	28 (77.8)	1.0 [Reference]		1.0 [Reference]	
Male	74 (43.5)	8 (22.2)	2.7 (1.2–6.5)		2.7 (0.9–9.7)	0.099
Age (years old)				0.001*		
< 10	13 (7.6)	9 (25.0)	1.0 [Reference]		1.0 [Reference]	
10 to 17	16 (9.4)	8 (22.2)	1.4 (0.4–4.8)		1.1 (0.2–6.5)	0.944
18 to 49	80 (46.7)	14 (38.9)	3.9 (1.4–11.0)		3.5 (0.7–17.8)	0.137
≥ 50	62 (36.3)	5 (13.9)	8.2 (2.4–31.5)		7.6 (1.5–42.7)	0.017*
Educational level				0.026*		
High school	31 (18.3)	12 (34.3)	1.0 [Reference]		1.0 [Reference]	
Elementary	113 (66.9)	15 (42.9)	2.9 (1.2–6.9)		2.7 (0.8–9.0)	0.111
Illiterate	25 (14.8)	8 (22.9)	1.2 (0.4–3.6)		0.11 (0.1–3.5)	0.576
Contact with soil				0.003*		
No	7 (4.17)	7 (20.6)	1.0 [Reference]		1.0 [Reference]	
Yes	161 (95.8)	27 (79.4)	5.9 (1.8–18.9)		4.4 (1.1–18.8)	0.038*
Drinking water source				0.043*		
River	22 (14.4)	1 (3.2)	1.0 [Reference]		1.0 [Reference]	
Artesian well	42 (27.5)	15 (48.4)	0.2 (0.01–0.8)		0.2 (0.01–1.0)	0.104
Springer water	89 (58.2)	15 (48.4)	0.3 (0.01–1.7)		0.4 (0.02–2.7)	0.455
Onicophagy				0.912		
No	121 (73.8)	26 (76.5)	1.0 [Reference]			
Yes	43 (26.2)	8 (23.5)	1.1 (0.5–2.9)			
Ingestion of raw meat				0.743		
No	154 (91.7)	34 (94.4)	1.0 [Reference]			
Yes	14 (8.33)	2 (5.56)	1.5 (0.4–10.4)			
Game meat consumption				0.581		
No	145 (85.8)	31 (91.2)	1.0 [Reference]			
Yes	24 (14.2)	3 (8.8)	1.6 (0.5–7.5)			
Owning a dog				0.472		
No	13 (7.8)	1 (2.9)	1.0 [Reference]			
Yes	153 (92.2)	33 (97.1)	0.4 (0.02–2.2)			
Owning a cat				1.0		
No	79 (47.6)	16 (47.1)	1.0 [Reference]			
Yes	87 (52.4)	18 (52.9)	1.0 (0.5–2.1)			
Owning a dog and a cat				1.0		
No	81 (48.8)	16 (47.1)	1.0 [Reference]			
Yes	85 (51.2)	18 (52.9)	0.9 (0.4–2.00)			

^*^Statistically significant

Area under the ROC curve (AUC = 0.823, 95% CI = 0.739–0.907) suggested that the final logistic regression model showed a moderate to excellent performance [39] (Additional file 1: Figure S1). No collinearity among variables was observed in the logistic regression model. All variables, except gender, presented inconsistent or missing data in the questionnaires; however they were maintained in the analysis since they incurred losses < 15.0%.

Overall, 186/208 (89.4%) quilombola individuals owned at least one dog (range: 1 to 10; average: 2.8) and 105/208 (50.5%) at least one cat (range: 1 to 10; average: 2.0); a total of 103/208 (49.5%) declared raising both. Most of the dogs (90/96; 93.8) were > 1 year old, according to their owners. During the visits, low body condition score was mostly observed in dogs. Lack of vaccine and deworming scheme was reported by all dog owners. *Toxocara* spp. eggs were detected in 5/96 (5.2%) dog fecal samples from quilombola communities (Table 4). The community with the highest prevalence of *Toxocara* spp. in dogs was Tronco (3/52; 5.8%), followed by Mamans (1/18; 5.5%) and Serra do Apon (1/21; 4.8%). No positive sample was verified in the Limitão community. Regarding hair samples, 2/96 (2.0%) dogs, both from Serra do Apon community, had *Toxocara* spp. eggs adhered to fur. One of these dogs presented viable *Toxocara* spp. eggs in hair from the dorsal (1 egg) and perineal (30 eggs) regions, while the other presented just one viable *Toxocara* spp. egg in the dorsal region. The presence of *Toxocara* spp. eggs in both feces and hair samples was observed in a dog. No *Toxocara* spp. eggs were recovered in dog hair samples from the others visited quilombola communities (Table 4).

**Table 4:** Frequency of *Toxocara* spp. eggs in fecal and hair samples of dogs living in quilombola communities of Paraná State, southern Brazil

Communities	Samples: positive /total (%)	
	Feces	Hair
Limitão	0/5 (0.0)	0/5 (0.0)
Mamans	1/18 (5.5)	0/18 (0.0)
Serra do Apon	1/21 (4.8)	2/21 (9.5)
Tronco	3/52 (5.8)	0/52 (0.0)
Total	5/96 (5.2)	2/96 (2.0)

A total of 18/60 (30.0%) soil samples were positive for *Toxocara* spp. eggs (Table 5). Herein, as the total number of recovered eggs was low (42 eggs), the average number of eggs per community was calculated considering 100 g of soil. The highest frequency of positive samples and number of recovered *Toxocara* spp. eggs were observed in quilombola community Mamans (7 eggs/100 g of soil), followed by Serra do Apon (4.5 eggs/100 g of soil), Limitão (1.5 egg/100 g of soil) and Tronco (1 egg/100 g of soil). Most of the *Toxocara* ssp. eggs recovered from soil were classified as non-viable (35/42; 83.3%) (Table 5).

**Table 5:** *Toxocara* spp. eggs recovered from soil samples collected in quilombola communities of Paraná State (southern Brazil) according to its morphological characteristic

Communities	Positive/total (%)	Morphological characteristics of *Toxocara* spp. eggs			
		V	NV	EM	E
Limitão	3/10 (30.0)	0	3	0	0
Mamans	10/20 (50.0)	3	24	0	1
Serra do Apon	3/10 (30.0)	2	7	0	0
Tronco	2/20 (10.0)	1	1	0	0
Total (%)	18/60 (30.0)	6/42 (14.3)	35/42 (83.3)	0	1/42 (2.4)

*V* viable, *NV* non-viable, *EM* embryonated egg, E: embryonated (containing larvae)

## Discussion

This is the first serosurvey to our knowledge based on a One Health approach that investigates the potential risk factors for toxocariasis in Brazilian quilombos along with environmental contamination and dog infection by *Toxocara* spp. Moreover, quilombola communities presented the highest seroprevalence (172/208; 82.7%) for toxocariasis in Brazil to date, surpassing a serosurvey in a rural adult population (247/344; 71.8%) of southern Brazil [12]. Living in rural areas itself is a major risk factor associated with seropositivity for *Toxocara* spp. (OR = 1.9) according to a global meta-analysis [11], corroborated by surveys in rural populations of Gabon, Africa (199/332; 53.6%) [40], and Thailand (101/132; 76.5%), Asia [41].

In addition to rural area exposure, higher toxocariasis seroprevalence is also more likely in those with lower educational and socioeconomic levels living under poor sanitary conditions as observed in our study [9]. Not surprisingly, a nationwide Brazilian survey has observed inadequate sanitation in 8291/8743 (94.8%) quilombola communities [16]. The main reported factors associated with quilombola vulnerability included low family income and education, difficulty in accessing drinking water, lack of health services [42–45], and food insecurity in the household [16, 46]. In this scenario, the lifestyle of quilombola populations favors high exposure to *Toxocara* spp. infection.

Aging remains controversial as an associated risk factor for toxocariasis. Logistic regression revealed age and contact with soil as predictive factors for toxocariasis, with a direct increase of seropositivity by age, corroborating a national USA serosurvey in which individuals > 50 years old were more likely seropositive than 6–11 year olds [47]. However, a study in a rural area in northern Brazil with 466 individuals from 5 to 90 years old showed higher seropositivity in 5 to 14 year old children (36.6%) than in older subjects (22.5%) [48]. In addition, higher toxocariasis seroprevalence was observed in younger subjects (10–19 years) followed by a decline in early adulthood and a second rise during older age in Jamaica [49]. This pattern in younger persons may be due to decreased exposure to infective *Toxocara* spp. stages with age and a subsequent decline in antibody levels over time, while an increase in prevalence at older age may be due to later exposure due to agricultural or other outdoor land activities [49].

As land subsistence practices and agricultural activities have been reportedly inherited in quilombola culture [16, 50], human infection risk may be related to close contact with soil contaminated by *Toxocara* spp. eggs in rural areas [11, 40], with all adult participants referring to agricultural practices in our study. Thus, labor activities of quilombola individuals may have favored a continuous exposure to infection via soil and, consequently, the maintenance of anti-*Toxocara* spp. antibodies levels over time. As expected in the present study, and reinforcing this statement, *Toxocara* spp. eggs were retrieved in soil samples of all four quilombola communities.

Male gender has been demonstrated as an associated risk factor for toxocariasis [47, 51, 52], with 1.3 increased odds of seropositivity in a meta-analysis study [11]. According to our results, both women and men were at risk of infection from ingestion of *Toxocara* spp. eggs via soil, probably from working in outdoor land activities and/or following poor hygienic habits.

Low educational level has been historically associated to toxocariasis [9], even in developed countries such as the USA [47, 51]. In Brazil, higher education was a protective factor against *Toxocara* spp. exposure (OR = 0.2) in persons experiencing homelessness [53]. Despite being considered a predictive factor for toxocariasis in univariate analysis but not in logistic regression, low educational level and socioeconomic status factors may concomitantly increase the risk of *Toxocara* spp. infection [9]. Likewise, univariate analysis here revealed an association with seropositivity and untreated water consumption (OR = 2.0), as source of drinking water has been considered a risk factor for *Toxocara* seropositivity [11]. As previously reported, untreated wastewater for irrigation was considered a contamination source of agricultural products with *Toxocara* spp. eggs in North Africa [54]. In addition, a systematic review included toxocariasis in the top list of water-borne transmitted parasitic diseases in the Middle East and North Africa, highlighting the importance of drinking water sources and sanitation facilities to reduce disease transmission [55]. No study on the persistence of *Toxocara* eggs in water has been found in Brazil or other Latin American countries.

Fecal and hair samples from the quilombola community dogs confirmed the presence of *Toxocara* spp. eggs. Although having dogs, cats, or both was not associated with human seropositivity, approximately 90% and 50% of quilombola individuals declared having at least one dog or one cat. Additionally, dogs and cats were mostly unleashed, free-roaming, and not dewormed in the studied quilombola communities. As contact with dogs and cats has already been considered another associated risk factor for toxocariasis, mainly in younger people [11, 56], companion animal deworming should be always recommended to reduce *Toxocara* spp. egg shedding into the environment and posterior transmission via soil ingestion to the exposed population [57].

In this study, no association was observed between seropositivity and ingestion of raw, undercooked, or game meat. The quilombola population in this study mostly (> 90.0%) reported no habits of consuming raw meat of either domestic or game animals, reducing the likelihood of toxocariasis via animal consumption. The ingestion of undercooked or raw meat from paratenic hosts, mainly cattle and chickens, has been associated with toxocariasis [58–60], mostly in Asian countries because of local habits of eating raw meat [61]. In Brazil, serosurveys and a review study [62] have indicated no association between consumption of raw meat and toxocariasis [13, 53, 63]. As previously shown for quilombola communities, although having low consumption of raw and undercooked meat, the logistic regression showed that those individuals who said they consumed raw or undercooked game meat were 2.4-fold more likely (*P* = 0.042, 95% CI = 1.1–5.9) to be seropositive for *Toxoplasma gondii* [64].

As limitations of our study, remote locations are difficult to access, restricting visits to quilombola communities. Thus, assessment of stool of quilombola individuals for potential infection by *Ascaris lumbricoides* was not possible. However, the protocol used herein for detecting anti-*Toxocara* spp. antibodies has been employed in other serosurveys evaluating people in vulnerabile conditions [13, 53], using pre-adsorption of sera with *A. suum* adult worm extract to mitigate cross-reactivity with other Ascaridia, including *A. lumbricoides* [26]. Additionally, the presence of *Toxocara* spp. in cat biological samples (hair and feces) was not analyzed because of refusal of quilombola communities due to health and cruelty risks when catching and restraining the animals. Furthermore, soil samples were collected from common areas used by the community, which may not fully reflect the individual exposure to contaminated soil with *Toxocara* spp. eggs. Finally, future studies should analyze soil samples from areas of land labor and include elderly subjects to fully establish *Toxocara* spp. in such quilombola communities.

## Conclusions

The high seroprevalence observed in quilombola communities of southern Brazil suggested high exposure to toxocariasis. The high vulnerability and close human-soil contact observed here as risk factors demand a One Health approach for detection, monitoring, and prevention of *Toxocara* spp. infection in both human and dog populations. Furthermore, educational improvement is necessary to prevent toxocariasis and other zoonotic infections.

### Supplementary Information

**Additional file 1: Figure S1.** Receiver-operating characteristic (ROC) curve [evaluating the accuracy of the multivariate logistic regression model (AUC = 0.823, 95% CI = 0.739–0.907)] to predict seropositivity for anti-*Toxocara* spp. antibodies in individuals of quilombola communities of Paraná State, southern Brazil

**Additional file 2: ** Raw data of associated risk factors of toxocariasis in quilombola communities of southern Brazil..

## Data Availability

All data generated or analysed during this study are included in this published article and its Additional file information.
