# Supplementary figures and images for: One health approach to toxocariasis in quilombola communities of southern Brazil
Source: Parasit Vectors. 2023 Oct 23;16:379. doi: 10.1186/s13071-023-06010-w (PMC10594776; doi:10.1186/s13071-023-06010-w)

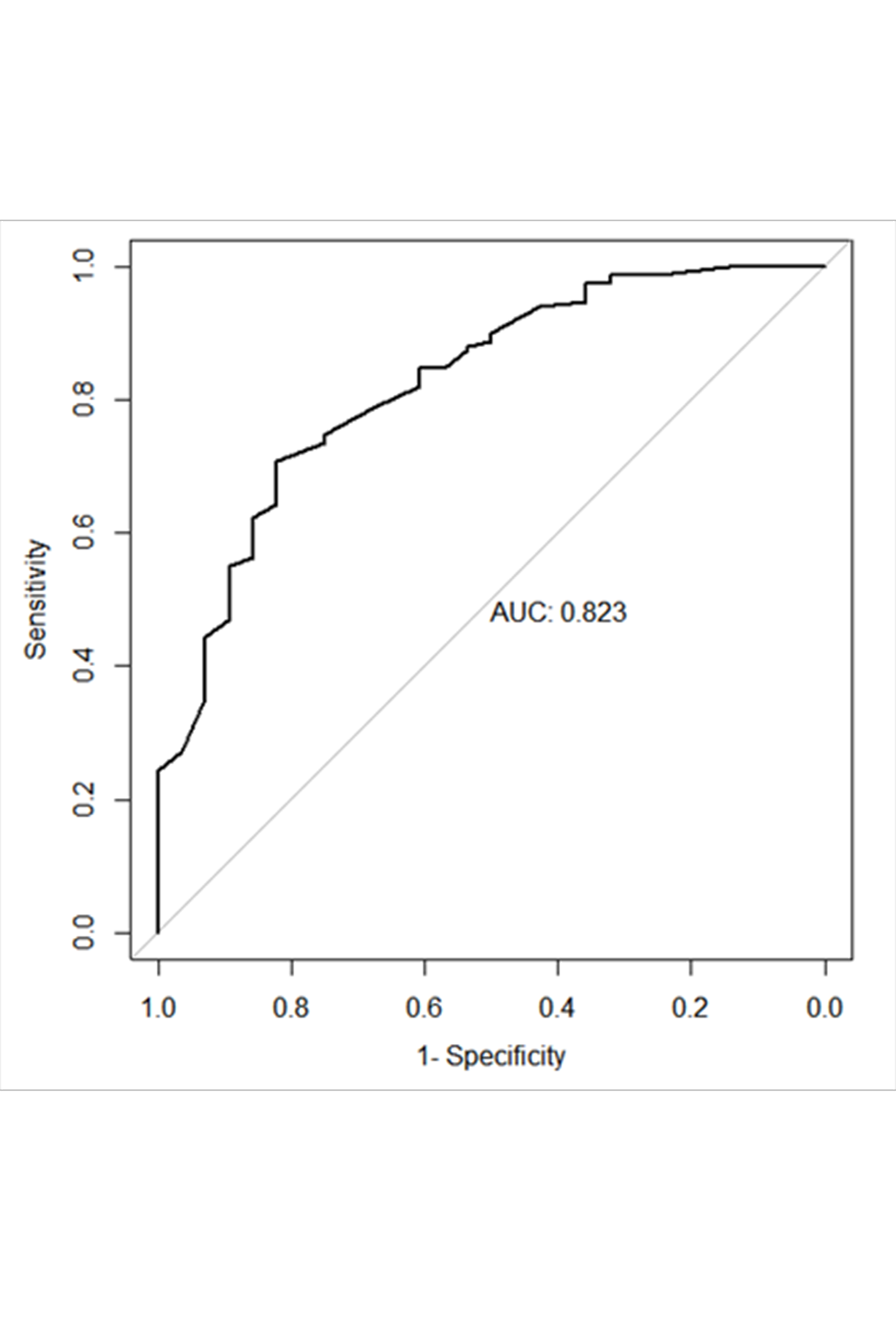

Supplement: Supplementary file 1 — Additional file 1: Figure S1. Receiver-operating characteristic (ROC) curve [evaluating the accuracy of the multivariate logistic regression model (AUC = 0.823, 95% CI = 0.739–0.907)] to predict seropositivity for anti-Toxocara spp. antibodies in individuals of quilombola communities of Paraná State, southern Brazil [file 13071_2023_6010_MOESM1_ESM.tif]
